# William Karesh: championing “One Health”

**DOI:** 10.2471/BLT.20.031020

**Published:** 2020-10-01

## Abstract

Preventing and responding to pandemics requires an integrated approach to human, animal and environmental health. William Karesh talks to Andréia Azevedo Soares.

Q: How did you become interested in the interface between human and animal health?

A: You could say it started with the animals. I grew up outside a small city in coastal South Carolina where there was a lot of wildlife. I would find orphaned baby animals and raise them. That turned into a passion that stayed with me through my education in biology and veterinary medicine. As for the interface, I think it just seemed obvious to me that all these different biological organisms, including us, are interconnected and that it makes sense to look at them as an ensemble. In the past two hundred years or so, the development of different medical specializations has discouraged cross-disciplinary thinking and collaboration. That seems problematic to me, given that the challenges we face tend to be composite. For example, antimicrobial resistance is at least as much about how we deal with wastewater and environmental contamination and the use of antibiotics in animals as it is about the way we consume or poorly prescribe antibiotics. So, you can only really tackle it by taking a composite or integrated approach.

Q: You coined the term "One Health" in 2003 in a Washington Post article. Can you explain what it means?

A: Basically, it’s the antithesis of the silo approach that I was just talking about. It is an intersectoral and interdisciplinary approach that focuses on where the health of humans, animals and environments or ecosystems converge. It is of particular relevance now because so many of the problems we face present interdisciplinary challenges. Antibiotics are one. Another is emerging zoonotic diseases like novel coronavirus disease (COVID-19). The precise origins of the novel coronavirus (SARS-CoV-2) have yet to be established, but it seems likely that it jumped from a wild animal into the human population at some point just as other coronaviruses have done for centuries. So, it would help to have some way of discussing the way these populations intersect in the ecosystem they share. About 75% of all emerging infectious diseases in humans are of zoonotic origin and that includes Ebola virus disease, influenza, severe acute respiratory syndrome (SARS) and Middle East respiratory syndrome (MERS).

And they have made their way into the human population for similar reasons – encroachment on natural habitats, consumption of wild animals, land use change, agricultural intensification driven by increased demand for animal protein, etc. These activities tend to encourage animal–human interactions which create opportunities for pathogens to jump across. Currently we, in the public health community, struggle to respond to these events because we deal with the outcome, i.e. the diseases, rather than the root causes of the outcome.

This happens because the group generating the risk – government ministries implementing economic development or agricultural policy that includes land clearance – are different to the group dealing with the outcome – those people implementing public health actions. The health ministry people may be very good at dealing with the outcomes, but they don't feel empowered to talk about land use change. So, they are left focusing on reactive response measures such as trying to reduce transmission within the human population or developing vaccines, but they can do little about the conditions that caused the pathogen to jump species in the first place. Ebola is a case in point, a perfect example of the tremendous costs incurred by only responding to outbreaks rather than implementing cross-disciplinary strategies to prevent them. We should be encouraging best practices in activities like mining and forestry to reduce exposure to high-risk animal sources and ensuring safe food supplies to reduce hunting for so-called bush meat. There should also be more effort to improve community engagement and education. In order to really address the problem and to improve our chances of preventing the next pandemic we need to work across disciplines and work better together, and that is the goal of the One Health approach. This is already happening with the tripartite collaboration between WHO, the Food and Agriculture Organization and the World Organisation for Animal Health, but we need to do much more.

Q: How can we achieve greater cross-disciplinary collaboration?

A: The starting point should be acknowledging the fact that no single organization can deal with the different components of the challenges we face. We need specialization but we need specialists to collaborate. So, we need to start by getting them to work together, peeling off pieces of the problem and then assembling groups with the appropriate expertise to address them. It happens all the time in the business world. Take the car industry: they bring together engineers, designers, risk mitigation specialists, finance people and marketing experts. They team up early and work towards a common goal to produce a product. We at the EcoHealth Alliance (EHA) use this as a standard operating approach.

“We need to work across disciplines.”

Q: Can you talk a little about how the EHA works?

A: The EHA is a relatively small organization but it engages with thousands of partners worldwide and brings together experts from multiple disciplines, including human health specialists, veterinarians, ecologists, anthropologists, mathematicians, economists and policy experts. As a group, we decide on the specific challenge we want to address, think about how we can bring in the additional groups we need and reach out to find local partners.

Q: Can you give a concrete example?

A: In South Africa, we have a large project which is focused on a vector-borne viral zoonosis called Rift Valley fever that occurs in periodic but severe outbreaks that impact people and livestock. Outbreaks are associated with periods of greater than average rainfall, but despite the different warning systems that exist, the outbreaks are not easy to predict. To inform risk mitigation and control measures you need to have a grasp of the environmental, animal health and human health factors that drive the epidemiological dynamics of the disease. So, we brought in experts from international and local weather services, the economic development board of South Africa, soil scientists to discover where diseases are more likely to occur, and botanists to see if certain kinds of flora support the mosquito vectors more than others. All to help inform risk-reduction strategies such as vaccination efforts or public outreach and education campaigns.

In another project we are talking with cold-storage facility experts. The cold-storage industry can play an important role in reducing the number of live animal markets, which, as has been seen with influenza, are a potential source of emerging diseases. I have been having conversations about the way cold storage can improve food supply chains in parts of the world where some of the major industry stakeholders have yet to establish a presence, the aim being to reduce the number of live animal markets and thus reduce the risk of a new emerging disease. We have our eyes on the road ahead rather than in the mirror.

Q: What do you mean by that?

A: Well, the traditional way of assessing the risk of a disease is to look at previous occurrences and put dots in a map and say, "these are the hotspots". But this is like looking at things in the rear-view mirror. The map shows where events happened before and not where they necessarily will happen in the future. The EHA approach is to look at the underlying characteristics of the places where these diseases occurred – the socioeconomic status, the annual rainfall, soil and vegetation coverage, education level, population density, the number of doctors/nurses/veterinarians per population – and then look for correlations between these characteristics and disease emergence. When we find, for example, the five most important factors that correlate, we find where these characteristics are present around the globe and thus indicate where in the future a new emerging disease is more likely to occur. In a nutshell, we map risk factors rather than the diseases themselves.

Q: What are the characteristics likely to be present in an emerging-disease hotspot?

A: Increased human population density, land-use change, including deforestation to free up land for agricultural production, changes in food production systems, and presence or contact with mammalian species.

Q: According to a new report by the United Nations Environment Programme and the International Livestock Research Institute, meat production has increased by 260% in 50 years and is one of the key drivers of environmental degradation. Should health authorities recommend diet changes as part of the One Health approach?

A: Yes. Meeting the challenge of sustainable nutrition is definitely something a One Health approach can help with. What are the drivers of the increased meat-eating trend? What is the impact? How can we address it? Education and public awareness, encouraging people to start making better choices is obviously part of the answer. But there are other ways to change behaviours, including interventions on the price of meat. Should the price of meat better reflect its true cost, i.e. including the cost of fresh water, subsidies for animal feed production or grazing land, forest loss, ground water pollution and methane release? There needs to be a thoughtful conversation about these externalities.

“The aim… is to engage far more of society.”

Q: Do you think the current crisis will act as a wake-up call, and lead to increased funding for pandemic prevention, perhaps based on One Health principles?

A: I hope so, but I am not sure. After the SARS outbreak we all thought there would be more investment and there was in the beginning. But then people went back to business as usual. And then H1N1 happened and we thought there would be more investment... The truth is that prevention is very hard to sell politically. I am still cautiously optimistic that this situation is going to change. I am hoping that new parts of society will get engaged, such as the private sector, educators, engineers, etc. While we should be proud of the advances made in the 20th century, we urgently need to welcome more people and broader skills to develop the next generation of approaches to solve the challenges of this century. The aim of One Health is to engage far more of society in finding creative solutions that simultaneously reduce the burden of disease, improve livelihoods and well-being, and protect our planet so that it can robustly sustain life for millennia.

